# Probing Dopant Size Effects on Defect Clustering and Vacancy Ordering in Lanthanide-doped Ceria

**DOI:** 10.1021/jacs.5c09862

**Published:** 2025-08-21

**Authors:** Jing Ming, Xingfan Zhang, Marzena Leszczyńska-Redek, Marcin Malys, Maciej Wojcik, Wojciech Wrobel, Stephen Hull, Franciszek Krok, Woongkyu Jee, Marcin Krynski, Alexey A. Sokol, Scott M. Woodley, C. Richard A. Catlow, Isaac Abrahams

**Affiliations:** † Department of Chemistry, 4617 Queen Mary University of London, Mile End Road, London E1 4NS, U.K.; ‡ Kathleen Lonsdale Materials Chemistry, Department of Chemistry, 4919 University College London, London WC1H 0AJ, U.K.; § Faculty of Physics, 49566 Warsaw University of Technology, Koszykowa 75, Warszawa 00-662, Poland; ∥ STFC ISIS Facility, Rutherford Appleton Laboratory, Chilton, Didcot, Oxon OX11 0QX, U.K.; ⊥ School of Chemistry, Cardiff University, Park Place, Cardiff CF10 1AT, U.K.

## Abstract

Dopant size is known to influence oxygen vacancy-mediated conduction pathways and ionic conductivity in doped ceria, yet the underlying atomic-scale mechanisms remain unclear. Here, we combine neutron total scattering and large-scale atomistic simulations to analyze the local defect structures of two representative doped ceria systems: Ce_0.8_Gd_0.2_O_1.9_ (GDC) and Ce_0.8_Nd_0.2_O_1.9_ (NDC). The local structure of GDC, a commercially used ion conductor, is investigated for the first time using neutron total scattering on ^160^Gd-enriched samples. GDC exhibits fewer defect clusters, with vacancy pairs preferentially aligned along ⟨111⟩ and ⟨110⟩ directions while disfavoring ⟨100⟩ direction within the cubic fluorite structure. The Gd–Gd interactions in GDC help destabilize ⟨100⟩ ordering, promoting a more open defect network that supports efficient oxygen-ion transport. Unlike Gd^3+^ (1.053 Å in 8-fold coordination with oxygen), the slightly larger dopant Nd^3+^ (1.109 Å) in NDC promotes a more compact defect configuration, characterized by increased defect clustering and stabilized ⟨100⟩ vacancy alignment due to dominant Nd–vacancy interactions, substantially reducing ionic conductivity. Gd^3+^ provides an optimal balance of lattice expansion and preserving favorable defect structure for ion transport. These findings provide a mechanistic understanding of dopant-size controlled conduction pathways in lanthanide-doped ceria and fundamentally contribute to the understanding of charge transport by ions, electrons, and protons in next-generation conducting materials.

## Graphical abstract

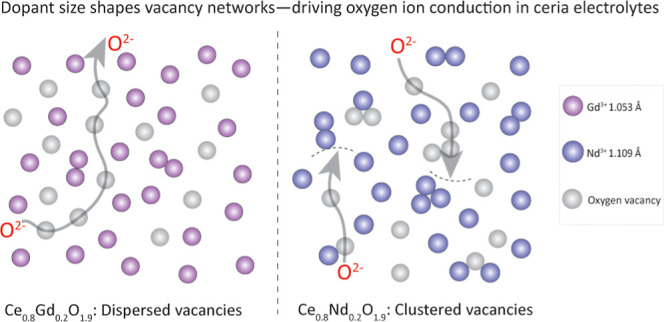

## 1 Introduction

Solid oxide fuel cells (SOFCs) provide a promising clean energy technology, offering high efficiency and low emissions through direct chemical-to-electrical energy conversion.^1,2^ Ceria-based materials, with their simple cubic fluorite structure, serve as highly efficient solid electrolytes in intermediate-temperature (500–700 °C) solid oxide fuel cells (IT-SOFCs).^3,4^ While pure ceria (CeO_2_) shows poor oxide ion conductivity due to its very low oxide ion vacancy (V_O_^••^) concentration under ambient conditions, vacancy concentration can be significantly increased by substitution of tetravalent Ce^4+^ by trivalent lanthanide (Ln^3+^) ions (eq 1).^5^

1 $$\frac{1}{2}{\mathrm{Ln}}_{2}O_{3}\overset{{CeO}_{2}}{\to }{\mathrm{Ln}}_{Ce}^{\prime }+\frac{1}{2}V_{O}^{\cdot \cdot }+\frac{2}{3}O_{O}^{\times }$$

Increases in oxygen vacancy concentration generally enhance the ionic conductivity of oxide-ion conducting electrolytes.^6^ However, the presence of defect clusters, as spatial groupings of dopants and oxygen vacancies, can strongly influence vacancy mobility.^7,8^ As dopant and vacancy concentration increase, isolated defects tend to form more clustered configurations, which can hinder ion transport and suppress ionic conductivity.^9^ For example, Murray et al.^10^ showed that in calculations on 14.3 mol % Y_2_O_3_ doped ceria, a reduction in ionic conductivity by 1.5 orders of magnitude is found when the Y^3+^ cations were ordered (Cu_3_Au type ordering) in the fluorite lattice compared to a fully random distribution, due to strong yttrium–vacancy association and fewer available migration pathways. Across various doped fluorites, including Y_2_O_3_-doped ZrO_2_ ^11^ and Sm^3+^-, Gd^3+^-, and Nd^3+^-doped CeO_2_ systems,^9^ a maximum in ionic conductivity occurs at a critical concentration (∼10–20 mol %), beyond which clustering suppresses conductivity.

Among the doped cerias, gadolinium-doped ceria (GDC, Ce_1–*x*_Gd_*x*_O_2–*x*/2_) and neodymium-doped ceria (NDC, Ce_1–*x*_Nd_*x*_O_2–*x*/2_) are both promising ion conductors. Gd^3+^ is the most practically used and best-established dopant among the lanthanide-doped ceria systems, providing excellent ionic conductivity and good phase stability,^1,3,12,13^ while, NDC has been reported to exhibit a particularly low minimum activation energy of 0.68 eV, lower than that of Gd^3+^-doped (0.70 eV), Sm^3+^-doped (0.72 eV), and Y^3+^-doped (0.78 eV) ceria, indicating highly promising bulk conductivity.^14,15^ However, GDC exhibits 2 to 3 times higher ionic conductivity than NDC at the same level of doping, even though Gd^3+^ and Nd^3+^ share similar chemical profiles.^8,14,16,17^ Butler et al.^18^ first attributed this observation to the location of Gd^3+^ at the minimum of the V_O_^••^–Ln_Ce_^*’*^ binding energy trend, resulting in a minimum impact on the migration of oxygen vacancies, and they proposed that the dopant size is a key parameter. However, the existing studies cannot fully explain how such a small difference in ionic radius (*r* = 1.053 Å and 1.109 Å in 8-fold coordination with O^2–^ for Gd^3+^ and Nd^3+^, respectively^19^) can account for such a large difference in conductivity. This requires a more detailed understanding of the local structural environments in both systems, which can be effectively probed using neutron-based techniques.^20–22^

Our recent neutron total scattering studies suggest that vacancy ordering may help explain the conductivity differences between these two systems, as Nd-doped ceria shows a preference for vacancy pairs aligned along the ⟨100⟩ direction.^23^ Although the ⟨100⟩ direction is a primary diffusion pathway in undoped and doped CeO_2_ due to its low migration barrier (0.5–0.6 eV in pure CeO_2_),^24^ it does not appear to correspond to the most energetically favorable vacancy alignment^9,16,25,26^ as the vacancy–vacancy repulsion is expected to be the greatest for this alignment. These observations raise a new question about the underlying structural and energetic drivers of this defect ordering, and whether such ordering could contribute to the difference in conductivity between these two systems. It is, however, a challenging problem both experimentally, due to the difficulty in accurate characterization of defect interactions at the atomic scale, particularly in Gd-doped ceria, where high neutron absorption of Gd hampers measurements,^27^ and theoretically, due to the complexity of exploring the vast configuration space of defect formation and interactions within the lattice of materials.^28–31^

Based on the concerns above, the overall aim of the present work is to determine whether a small difference in dopant size can lead to a significant conductivity enhancement in such systems. The use of isotopically enriched ^160^Gd, which avoids high neutron absorption,^27,32^ allows for previously inaccessible local structural details in GDC to be accessed. By combining neutron total scattering data and theoretical defect calculations, this study enables a detailed comparison of GDC and NDC at the atomic scale. This integrative approach has the potential to reveal how subtle dopant variations influence defect structures and conduction pathways at the atomic scale. Such insights could provide a mechanistic foundation for tuning ionic transport in doped fluorites and may help guide the development of next-generation solid electrolytes.

## 2 Methods

### 2.1 Materials Synthesis

A dopant concentration of 20% (*x* = 0.2) in the system of Ce_1–*x*_Gd_*x*_O_2–*x*/2_ (GDC) was chosen as a representative composition near the upper boundary of the optimal conductivity range for GDC (typically 10–20 mol %) and this composition is widely used in the literature.^8,9,33–35^ In our previous study on NDC, the *x* = 0.2 composition was found to be representative of the Ce_1–*x*_Nd_*x*_O_2*x*/2_ system across the dopant range from *x* = 0.05 to 0.3.^23^ At these compositions, defect concentrations are sufficiently high to be detected using neutron scattering methods, enabling a meaningful comparison of defect structures between Gd^3+^ and Nd^3+^. For structural studies, Ce_0.8_Gd_0.2_O_1.9_ was synthesized via solid-state reaction using CeO_2_ (Aldrich, 99.9%) and ^160^Gd_2_O_3_ (BuyIsotope, enrichment 97.7%). Stoichiometric molar ratios of these oxides were mechanically ground in a planetary ball mill using ethanol as a dispersant for *ca*. 24 h. The dried precursors were then calcined at 1550 °C for 30 h. The requirement for ^160^Gd isotopically enriched samples for neutron studies, limited synthesis to a solid-state route, as the precursor was only readily available in oxide form. All experimental and RMC results for Ce_0.8_Nd_0.2_O_1.9_ (NDC) are derived from our previous study and shown for comparison.^23^

For electrical measurements, it is helpful to note here that there are some discrepancies in reported conductivity values across different studies attributed to variations in sample synthesis, sintering, particle distribution and densification.^9,36,37^ However, the conductivity of GDC is widely reported to be higher than that of NDC by most of the studies.^3,8,17^ To minimize these effects, samples were prepared by a wet-chemical coprecipitation route, which enables better control over powder reactivity and facilitates sintering into dense ceramics with lower porosity for reliable conductivity values. In the coprecipitation route, the nitrates Ce­(NO_3_)_3_·6H_2_O (Aldrich, 99%), Gd­(NO_3_)_3_·6H_2_O (Aldrich, 99.9%) or Nd­(NO_3_)_3_·6H_2_O (Aldrich, 99.9%) were dissolved in distilled water (yielding a concentration of approximately 1 M metal nitrate). A 0.05 M oxalic acid solution was prepared from H_2_C_2_O_4_·2H_2_O (Aldrich, ≥99%) and distilled water. An aqueous ammonia solution (28–30% NH_3_, Supelco) was then added dropwise to neutralize the solution (pH ∼7). In each case, the metal nitrate solution was then added dropwise to precipitate the metal oxalate. The resulting precipitate was filtered, rinsed with distilled water, dried, and calcined at 700 °C for 1 h in a platinum crucible. After cooling, the calcined powder was mixed with polyethylene glycol (Alfa Aesar) and pressed into pellets (10 mm diameter, 2–3 mm thickness) using a uniaxial press, followed by isostatic pressing at 400 MPa. The pellets were sintered at 1200 °C for 5 h and slowly cooled down to room temperature in the furnace over *ca*. 10 h. They were then cut into rectangular blocks (5 × 3 × 2 mm^3^) using a diamond saw for impedance spectroscopy measurements. Each polished surface was coated with platinum electrodes via cathodic sputtering.

### 2.2 Materials Characterization

#### 2.2.1 Electrical Measurements

Electrical behavior was investigated by a.c. impedance spectroscopy using a fully automated Solartron 1255 analyzer in conjunction with a bespoke automatic current/voltage converter in the frequency range from 10^–1^ to 10^6^ Hz. Impedance spectra for each composition were collected over two cycles of heating and cooling ramps at selected stabilized temperatures from *ca*. 200 to 830 °C in 20 °C increments. The Nyquist plots of impedance spectra for GDC and NDC at selected temperatures are presented in Figure S3 of the Supporting Information. Both materials exhibit similar profiles across the measured temperatures. In all cases, only a portion of the full impedance spectrum is visible, as most processes within the grains and grain boundaries fall outside the measurement frequency window. Therefore, only the total resistance (*R*_t_), as indicated by the arrows on the plots, could be determined. In panels (a,b) of Figure S3, both GDC and NDC exhibit a distinct and depressed semicircle, indicating high total resistance, with GDC showing lower and less obvious resistances than NDC. The pronounced tail extension at lower frequencies is indicative of polarization of the electrolyte/blocking electrode interface, while at intermediate and high temperatures, only part of impedance spectra related to the electrode is visible (Figure S3c–f).

The density of sintered pellets of GDC and NDC was measured using the Archimedes principle by displacement of isobutanol. Both pellets were found to have relative densities of over 95% (95.5% and 97.5% for GDC and NDC, respectively) of their theoretical values.

#### 2.2.2 X-ray Powder Diffraction (XRD)

Phase purity and crystal structure were examined using X-ray Powder diffraction (XRD) at room temperature using Ni-filtered Cu–Kα radiation (λ = 1.5418 Å) on a PANalytical Empyrean diffractometer fitted with a PIXcel-3D detector. Data were collected in flat-plate Bragg–Brentano geometry over the 2θ range 5–125°, in steps of 0.01313°, with an effective count time of 250 s per step.

#### 2.2.3 Neutron Diffraction

Powder neutron diffraction data were collected at room temperature on a powdered sample of Ce_0.8_^160^Gd_0.2_O_1.9_ on the Polaris diffractometer at the ISIS Facility, Rutherford Appleton Laboratory, UK. The sample was contained in a 6 mm diameter cylindrical vanadium can (wall thickness of ca. 0.02 mm) located in front of the back scattering detectors. A data set corresponding to 2200 μA h of proton beam current was collected, as well as data for an empty vanadium can (2000 μA h), the empty instrument (1960 μA h) and a 5 mm diameter vanadium rod (1750 μA h) for data correction and normalization.

The total neutron scattering data from five detector banks were used: back scattering (average angle 146.72°), 90° (average angle 92.59°), intermediate-angle (average angle 52.21°), low-angle (average angle 25.99°) and very -low- angle (average angle 10.40°) detector banks, covering *d*-spacing ranges of approximately 0.04–2.6 Å, 0.05–4.1 Å, 0.73–7.0 Å, 0.13–13.8 Å and 0.3–48 Å, respectively. The data were then summed, corrected and normalized using the GudrunN software.^38^

#### 2.2.4 Rietveld Refinement and Total Scattering Analysis

Average structural analysis was carried out by the Rietveld method using the GSAS suite of programs^39^ via the EXPGUI interface,^40^ with a combination of neutron (back scattering and 90° detector banks) and X-ray diffraction (XRD) data. The initial model was based on a standard fluorite structure in space group *Fm*3̅*m*. The neutron scattering length for Gd was calculated assuming *ca*. 2% residual naturally abundant Gd in the isotopically enriched ^160^Gd sample. The refined oxygen site occupancy was close to the theoretical value (0.95), assuming Ce and Gd to be in the +4 and +3 oxidation states, respectively. The fitted diffraction patterns are presented in Figure S1, with crystal and refinement parameters detailed in Table S1, as well as the refined structural parameters provided in Table S2 of the Supporting Information.

For total scattering analysis, the RMC method was used to fit the normalized total scattering structure factor, *S*(*Q*), and total pair correlation function, *G*(*r*), using the RMCProfile software.^41–43^ Differential correlation function *D*(*r*) and bond valence sums (BVS)^44^ were used as weak constraints to ensure that long-range ordered and electrostatically realistic models were achieved, respectively. The initial configurations for the RMC analysis consisted of 10 × 10 × 10 supercells of the cubic crystallographic cell based on the average crystal structure determined by Rietveld analysis, with 11,600 atoms and 400 oxide ion vacancies, the latter specifically defined as a null scattering atom. To ensure statistical significance, 10 parallel sets of calculations were performed, each with different random distributions of Ce/Gd atoms and oxygen atoms/vacancies (O/V_O_^••^) in positions corresponding to the 4*a* and 8*c* sites in the crystallographic cell, respectively, ensuring that the stoichiometry of the composition was maintained. Ce/Gd and O/V_O_^••^ swapping was performed throughout the calculations to ensure no bias from the starting configurations was maintained in the final configurations. Ten parallel sets of calculations were also performed without atom swapping for comparison. Calculations were carried out over 5 days, corresponding to over 10 × 10^7^ moves. The fitted S­(*Q*) (with detail of the fit at low *Q* inset) and *D*(*r*) profiles for a representative configuration are shown in Figure S2.

### 2.3 Theoretical Calculations

#### 2.3.1 Shell-Model Interatomic Potential Techniques

Doped ceria is a highly complex system for computational modeling due to the existence of interacting defects, substantial lattice distortions, and the vast configurational space associated with different dopant–vacancy arrangements. A systematic investigation of such systems requires atomistic models that are sufficiently large to ensure accuracy, while also maintaining computational efficiency to enable exploration of various defect configurations. Modeling defect complexes under periodic boundary conditions requires a very large supercell (over hundreds of atoms) to minimize the errors caused by spurious image–image interactions. Moreover, conventional generalized gradient approximation (GGA) density functional theory (DFT) functionals, with or without Hubbard U correction, are known to be insufficient to predict accurate formation energies for oxygen vacancies in CeO_2_.^45^ While hybrid DFT functionals yield sufficient accuracy, their computational cost makes them impractical for studying thousands of distinct defect configurations. In contrast, the Mott–Littleton approach^46^ avoids the artifacts of periodic boundary conditions by treating long-range electrostatics analytically, effectively simulating isolated defects in an infinite dielectric medium. Our previous studies have shown that, within the Mott–Littleton approach, our developed shell-model potentials accurately reproduce defect structures and formation energies in close agreement with hybrid DFT results, surpassing the accuracy of DFT + *U* predictions.^24^ Therefore, our approaches not only enable accurate simulation of charged defects within large atomic models, but also provide computational efficiency for the systematic investigation of thousands of distinct defect cluster configurations in doped ceria.

The shell model (Figure 1a) proposed by Dick and Overhauser is a powerful theoretical framework to simulate the structure and properties of defects in ionic solids.^47^ This model overcomes the limitations of simpler interatomic potentials (such as the rigid model) by incorporating ionic polarizabilities into simulations, which are essential for accurately modeling the dielectric properties of ionic crystals as well as defect- and impurity-induced electronic polarization. In this model, each ion is represented by a core, corresponding to the nucleus and nonpolarizable electrons, and a shell, representing the polarizable electron cloud. The sum of the core and shell charges is typically the formal ionic charge. The core and shell of each ion are connected via a harmonic potential. Electrostatic interactions are calculated using Coulombic potentials, while short-range and dispersion interactions are parameterized with fitted potentials.

**Figure 1:**
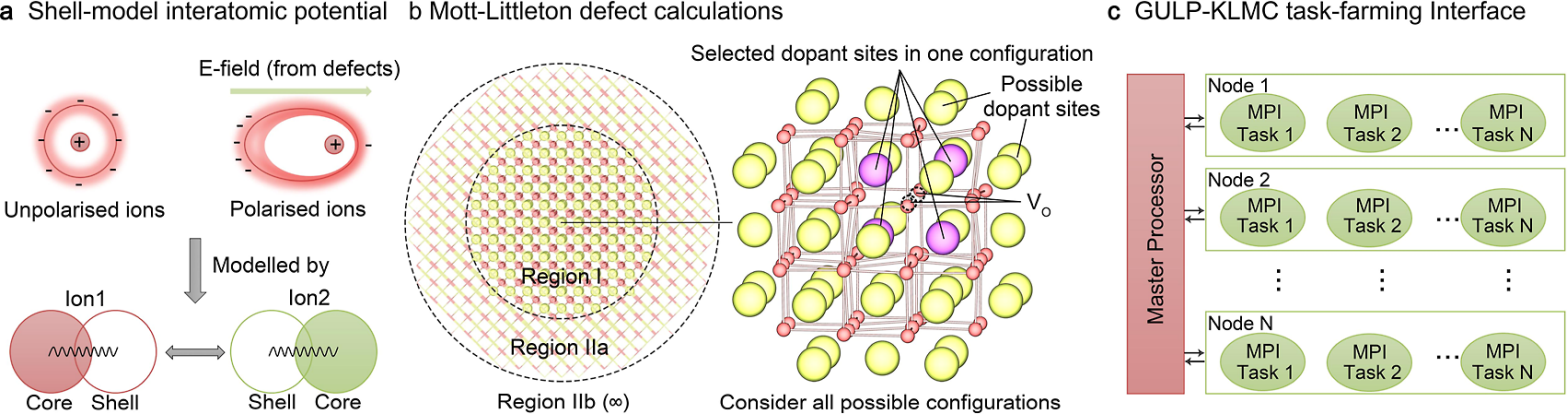
Computational techniques and simulation models for studying doped ceria. (a) Shell-model interatomic potential techniques. (b) The Mott–Littleton approach for modeling the formation of point defects and defect clusters at the dilute limit. (c) A GULP–KLMC task-farming interface for high-throughput calculations of complex systems with a vast configurational space.

In this study, we employed our recently developed shell-model potentials for CeO_2_, which reproduce a wide range of physical properties of CeO_2_ (and CeO_2–*x*_), especially the structures and formation energies of defects.^24,48^ The Gd–O interactions were adapted from the previous work of Butler et al.,^18^ used for studying Gd-doped ceria. Additionally, in this work, a new interatomic potential for modeling Nd–O interactions was developed based on the structural and physical properties of Nd_2_O_3_. The performance of this potential in reproducing the structures and properties of C-Nd_2_O_3_ and A-Nd_2_O_3_ is shown in Table S5. The consistency between the predictions of the shell-model potential and experimental as well as DFT reference data demonstrates the reliability of the simulation results. A format readable by the General Utility Lattice Program (GULP) code, discussed below, of the whole set of potentials used in this work is also provided in Table S6 of the Supporting Information.

All calculations based on interatomic potentials, including the fitting of new potentials, were conducted using the GULP code.^49,50^ To enhance the computational efficiency of various possible configurations of defect complexes, a recently developed KLMC–GULP task-farming interface (Figure 1c) was used.^51^ This interface supports high-throughput parallel execution of large numbers of GULP calculations on high-performance computing (HPC) clusters, leveraging both task farming and the Message Passing Interface (MPI) features.

#### 2.3.2 Mott–Littleton Defect Calculations

Theoretical calculations of defect formation and interactions at the dilute limit were performed using the Mott–Littleton approach (Figure 1b).^46^ Charged defects or complex defect clusters in ionic solids involve the polarization and displacement of a substantial number of atoms. The Mott–Littleton approach, alongside other nonperiodic embedded-cluster methods including the hybrid QM/MM embedded-cluster method, inherently avoids periodic image–image interactions intrinsic to supercell models, thereby accurately modeling localized states in solids.^24^ The Mott–Littleton approach partitions a crystal model into two regions. Region I is a central area containing a point defect or defect cluster, explicitly allowing for ionic relaxation and shell polarization. The outer regions, denoted as IIa and IIb, represent the electrostatic environment of the infinite solid, which are treated by harmonic approximation and macroscopic dielectric response, respectively.

#### 2.3.3 High-Throughput Workflow for Modeling Defect Clusters

In this work, a high-throughput Python-based workflow was developed to systematically investigatethe relative stability of different dopant configurations in ceria associated with the formation of oxygen vacancy pairs aligned along the ⟨100⟩, ⟨110⟩, and ⟨111⟩ crystallographic orientations. The cutoff radius for Region I was set to 15 Å, including approximately 1000 active atoms in the structural relaxation, and 30 Å for Region IIa. Dopant ions were introduced in the nearest-neighbor (NN) and next-nearest-neighbor (NNN) sites of the two vacancies as potential substitutional positions, exploring all possible combinations. For example, in the case of ⟨111⟩-aligned vacancy pairs, configurations were generated for systems with 1, 2, 3, and 4 dopants, corresponding to 25, 300, 2,300, and 12,650 different atomic arrangements, respectively. Then, high-throughput calculations were performed using the KLMC–GULP task-farming interface on the ARCHER2 HPC facility. The calculations included full atomic relaxation surrounding the defect structures, allowing the determination of relative defect energies corresponding to different dopant distributions. Next, the relaxed structures were subjected to a postprocessing step to evaluate the atomic displacements of the two vacancies. Configurations in which oxygen migration occurred (defined as any oxygen atom moving more than 2 Å during the relaxation process) were discarded, as such large displacements disrupted the intended vacancy ordering; other configurations were retained for further analysis. The retained configurations were subsequently analyzed to determine key properties, including their relative stability, on-site electrostatic (Madelung) potentials, and temperature effects, as discussed below. This workflow facilitated a systematic investigation of the interplay between dopant configurations and defect behavior in ionic solids, providing theoretical insights into the stabilization mechanisms of ordered oxygen vacancies in doped ceria as observed in experiments.

#### 2.3.4 Formation Energies of Defect Complexes

The formation of the [2 V_O_^••^–4Ln_Ce_^*’*^]^×^ defect complex is described in eq 2. In the charge-neutral case, the introduction of four substitutional dopants at the cerium sites in CeO_2_, Ln_Ce_^*’*^, is compensated by the formation of two doubly charged oxygen vacancies (V_O_^••^).

2 $$2{\mathrm{Ln}}_{2}O_{3}+4{Ce}_{Ce}^{\times }+2O_{O}^{\times }↔{\left[2V_{o}^{\cdot \cdot }-4{\mathrm{Ln}}_{Ce}^{\prime }\right]}^{\times }+4{CeO}_{2}$$

The formation energy of such defect complexes can be calculated by

3 $$E_{form}=E_{defect}+4{\Delta E}_{L}\left({CeO}_{2}\right)-2\Delta E_{L}\left({\mathrm{Ln}}_{2}O_{3}\right)$$

where *E*_defect_ is the calculated Mott–Littleton defect energy of the [2V_O_^••^–4Ln_Ce_^*’*^]^×^ complex. The calculated lattice energies of these oxides from interatomic potentials, Δ*E*_L_(CeO_2_) = −107.50 eV, Δ*E*_L_ (*C*-Nd_2_O_3_) = −129.26 eV, and Δ*E*_L_ (*C*-Gd_2_ O_3_) = −132.68 eV, were employed. A lower formation energy of the defect complex indicates a more energetically favorable configuration.^52^

#### 2.3.5 Temperature Effects

We also employed statistical methods within the canonical ensemble (*NVT*) to calculate the statistically averaged contributions of each defect configuration to the macroscopic defect behavior under different temperatures. The probability (*P*_*i*_) of the formation of each defect configuration under a given temperature *T* is given by

4 $$P_{i}=\frac{\exp\left(-\frac{E_{i}-E_{\min}}{k_{B}T}\right)}{\sum_{i}\exp\left(-\frac{E_{i}-E_{\min}}{k_{B}T}\right)}$$

here, *E*_*i*_ is the Mott–Littleton defect energy of a particular configuration *i*, *E*_min_ denotes the lowest defect energy among all possible configurations in the ensemble, and *k*_B_ is the Boltzmann constant. This equation gives the normalized probability of finding any possible configuration based on the calculated defect energy. Configurations with lower energies have higher probabilities of formation, especially at lower temperatures; while with increasing temperature, the contributions from less stable states become more significant.^53^ The macroscopic statistically averaged defect energy was determined by summing the weighted energies of all possible states

5 $$\mathrm{E̅}=\sum_{i}E_{i}P_{i}$$

#### 2.3.6 On-Site Electrostatic Potential

The Madelung potential (V_Mad_^O^) at the oxygen (O) sites was calculated using

6 $$V_{Mad}^{O}=k_{e}\sum_{ions}\frac{q_{ions}}{r_{o‐ions}}$$

where *k*_e_ is the dimensional Coulomb constant, *q*_ions_ is the charge of a surrounding ion, and *r*_o‑ions_ stands for the distance between the surrounding ion and O.

## 3 Results and Discussion

### 3.1 Ionic Conductivity

Figure 2a shows Arrhenius plots of total conductivity for the *x* = 0.2 composition of GDC compared to that of NDC at the same level of substitution. Deviations from ideal behavior are observed between 500 and 600 °C, with the activation energies above this temperature range lower than below it. This difference in low- and high-temperature activation energies has previously been attributed to oxide ion vacancy trapping through dopant–vacancy association, a phenomenon more prevalent at lower temperatures.^14,15,37,54^ Consistent with this, our previous study on NDC found that while dopant–vacancy association was still evident at 600 °C, it was markedly reduced compared to the strong association observed at room temperature.^23^

**Figure 2:**
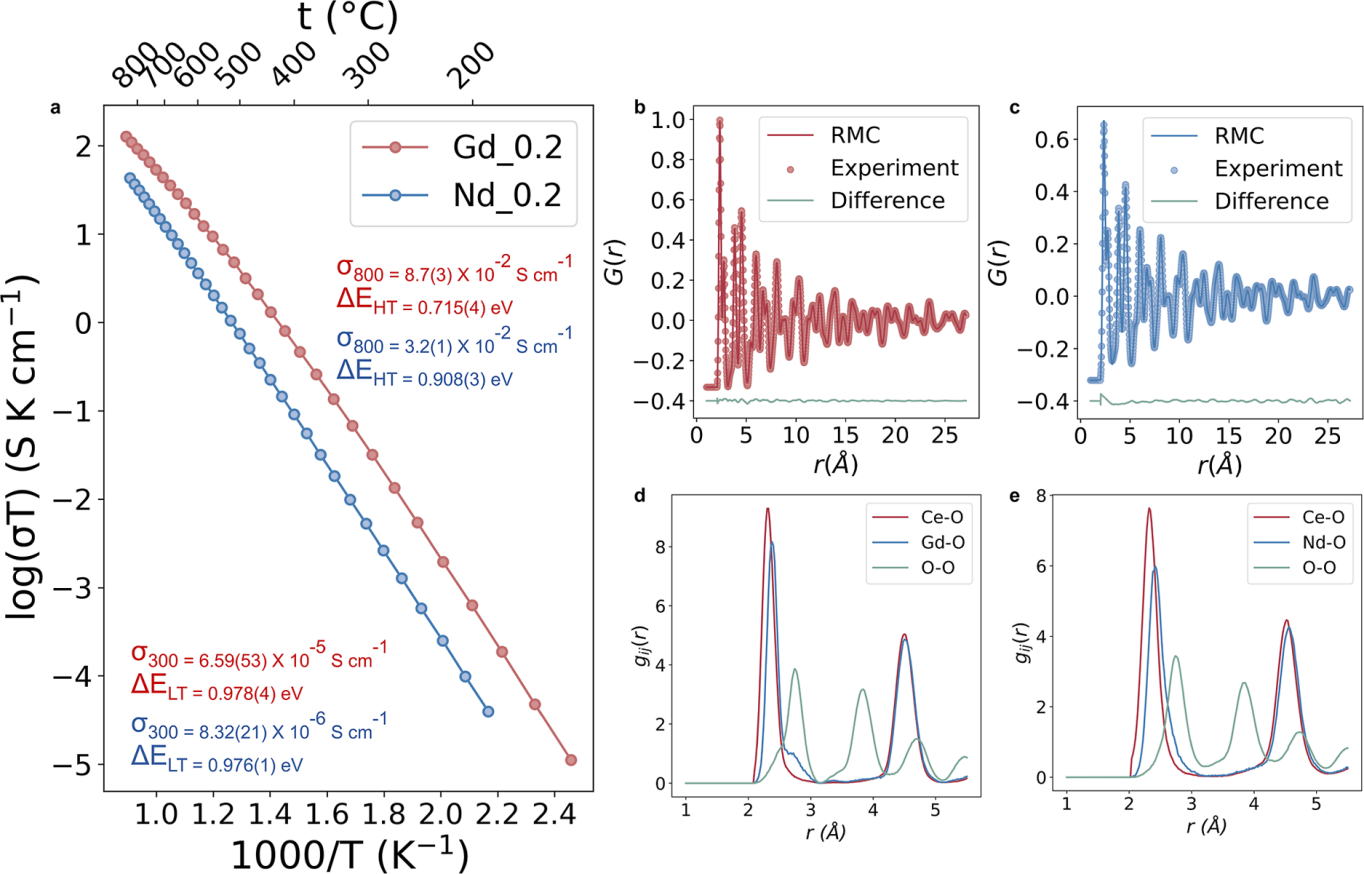
(a) Arrhenius plots of total conductivity for GDC (red) and NDC (blue), collected from the 2nd cooling ramp, showing conductivity and corresponding activation energies across both high-temperature (HT, 800 °C) and low-temperature (LT, 300 °C) regimes. (b,c) RMC-fitted normalized total pair correlation function G­(*r*). (d,e) Selected partial pair correlations *g*_*ij*_(*r*) for GDC and NDC; the NDC data are adapted from ref 23 for comparison.

GDC consistently outperforms NDC, showing higher ionic conductivity across the entire temperature range. Owing to practical constraints, the samples for conductivity and neutron studies were prepared by different synthetic routes, which may slightly influence absolute conductivity values due to the difference in microstructure and density,^34,37^ but do not alter the observed trend. This trend of our measurements agrees with broader findings reported in earlier studies, which identified Gd-doped ceria as exhibiting the highest ionic conductivity among all lanthanide-doped ceria across a wide temperature range, spanning both dilute regimes^55^ and at higher doping levels.^13^ While NDC demonstrates reasonable conductivity, its overall performance remains lower than that of GDC, in agreement with literature values.^14^ The observed conductivity differences of GDC and NDC can be understood from the defect chemistry of doped ceria systems. As discussed earlier, Gd is widely regarded as an optimal dopant due to its balance between ionic radius and minimal lattice distortion, resulting in the weakest dopant–vacancy interactions among the lanthanides.^18,56^ In the following section, we investigate the local structural environments in GDC and NDC using total scattering techniques, aiming to reveal how atomic-scale features may shape their conduction pathways.

### 3.2 Crystal Structure

The Rietveld-fitted diffraction patterns (Figure S1) confirm a cubic fluorite structure (*Fm* 3®*m*) for GDC, consistent with its standard diffraction pattern (ICSD-182976)^57^ and with no indication of secondary phases. The quality of the fits from RMC modeling to the G­(*r*) profile for GDC (Figure 2b) confirms a high level of agreement with the neutron total scattering data, indicating that the overall structure is well-reproduced, with no significant differences compared to the fit shown for NDC in Figure 2c. Selected partial pair correlation functions *g*_*ij*_(*r*) in the GDC and NDC systems are shown in Figure 2d,e. The mode (the first peak maximum, most probable distance) and mean (the weighted average) contact distances within the first coordination shell are derived from the g_*ij*_(*r*) profiles across 10 parallel runs and are summarized in Table 1. The Ce–O and Gd–O mode distances are consistent with values using K-edge extended X-ray absorption fine structure (EXAFS) measurements.^58,59^ The two systems show very similar profiles, with the mean metal oxygen distances slightly longer in the Nd-doped system. Mean distances are generally longer than the mode distances due to asymmetry in the pair distribution. The weighted average values of the mean distances are closer to the bond distances obtained from the Rietveld analysis, especially in the case of GDC.

**Table 1:** Mode and Mean Metal–Oxygen Distances (Å) Derived From Final RMC Configurations Compared to Those From Rietveld Analysis in Ce_0.8_Gd_0.2_O_1.9_ and Ce_0.8_Nd_0.2_O_1.9_ ^a^

composition	Ce_0.8_Gd_0.2_O_1.9_		Ce_0.8_Nd_0.2_O_1.9_	
type	mode	mean	mode	mean
Ce–O	2.300(1)	2.3537(5)	2.300(3)	2.373(1)
Gd/Nd–O	2.350(2)	2.4593 (12)	2.370(9)	2.497(3)
M–O (weighted)	2.3098(16)	2.3631(8)	2.314(3)	2.398(1)
Rietveld	2.34942(1)		2.35981(1)	

a Values are averaged over 10 parallel configurations, with standard deviations given in parentheses. Data for Ce_0.8_Nd_0.2_O_1.9_ are taken from ref 23.

### 3.3 Defect Clustering

Figure 3a,d,g illustrate the Gd and V_O_^••^ distributions in GDC, derived from a representative final RMC configuration. These projections highlight the key local structural features that emerge in GDC, which are further quantified by the nearest neighbor “coordination numbers” (Table S3) and corresponding percentages for different pairs (Table S4). Oxide ion vacancies near Gd^3+^ cations form Gd–V_O_^••^ associations (Figure 3a), with an 8.3% occurrence, moderately above the 5% expected for a fully random distribution. This suggests that while such small associations exist, they do not strongly limit vacancy mobility. Clustered oxide ion vacancies, represented by groups of 2 or 3 gray dots with gray links (Figure 3d), exhibit a 9.7% occurrence rate, also slightly elevated from a random distribution, yet still indicative of a well-dispersed vacancy network with minimal clustering. Additionally, the clustering of Gd^3+^ ions is represented by two or three closely situated purple dots with pink links (Figure 3g). Such Gd^3+^–Gd^3+^ nearest-neighbor pairs account for 24% of cation neighbors around Gd, compared to a value of 20% for a simple random distribution, indicating a slight tendency for dopant cations to form local associations. This tendency for dopant clustering aligns with the structural nanodomains often seen in transmission electron microscopy studies of GDC,^60^ and with previous EXAFS reports showing that Gd^3+^ exhibits weaker dopant clustering tendencies than La^3+^ and Y^3+^, which tend to form dopant-rich clusters.^59^

**Figure 3:**
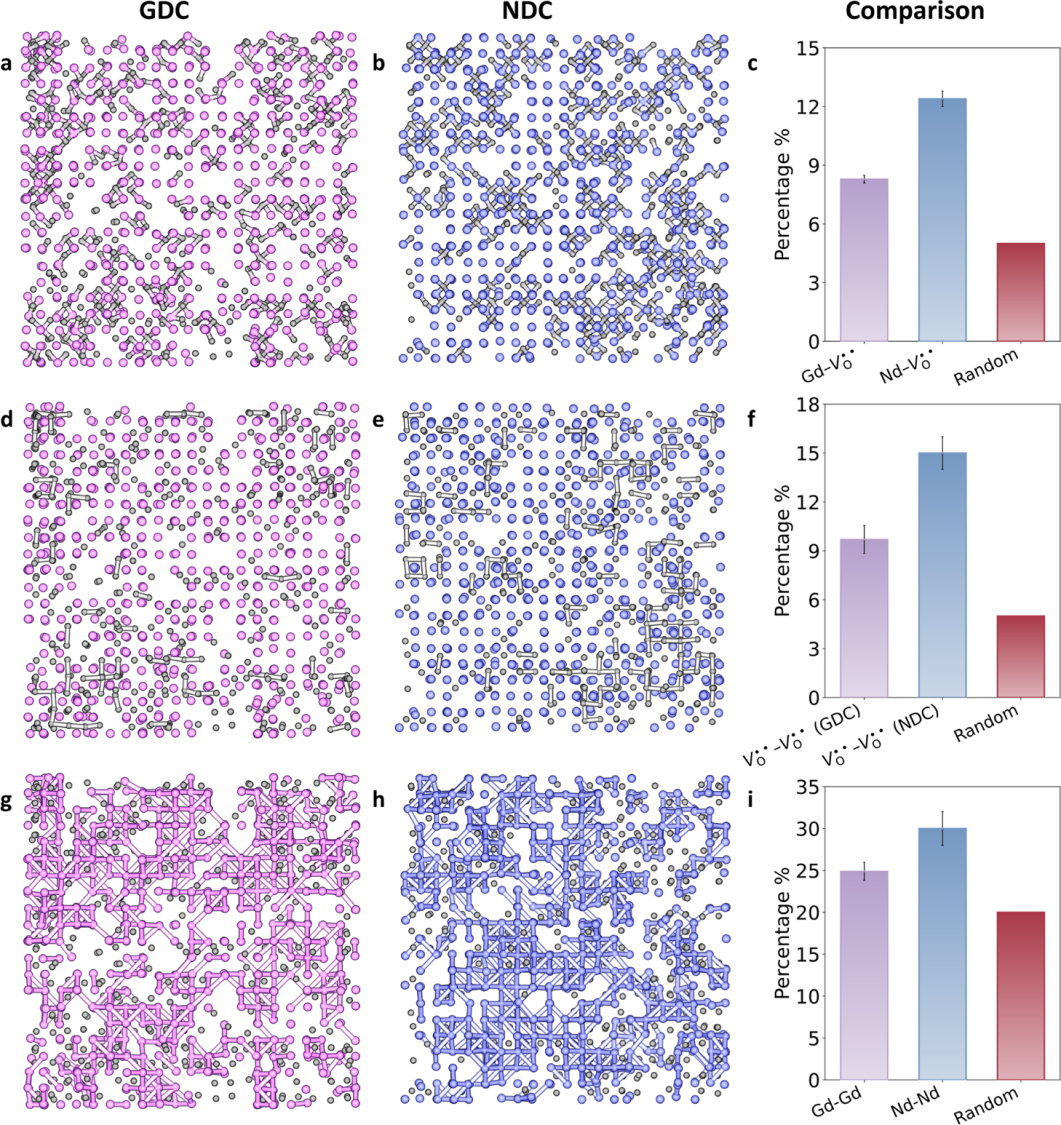
Local structural features derived from final RMC configurations in GDC and NDC, pink, blue, and gray spheres represent Gd, Nd, and V_O_^••^, respectively. Panels (a,b) show Ln–V_O_^••^ pair distributions, (d,e) depict V_O_^••^–V_O_^••^ distributions, and (g,h) represent Ln–Ln pair distributions in GDC and NDC, respectively, with nearest-neighbor contacts highlighted. Bar charts in c, f and i quantitatively compare the percentage (%) of nearest-neighbor distributions for Ln–V_O_^••^, V_O_^••^–V_O_^••^, and Ln–Ln pairs, respectively, against fully random distributions (Random). The NDC data are adapted from ref 23.

For comparison, Figure 3b, e and h show Nd and V_O_^••^ distributions in NDC, based on a representative final RMC configuration from work described previously,^23^ with differences in defect clustering between GDC and NDC summarized in Figure 3c,f,i. Unlike GDC, NDC exhibits significantly higher defect clustering across all metrics, reaching two to three times to the respective random distribution values (Table S4) and consistently exceeding those observed in GDC. The more frequent dopant–vacancy interactions in NDC are indicative of stronger defect clustering that would be expected to result in vacancy immobilization.

The present results show that in GDC, vacancy distributions remain largely random despite the presence of minor local associations. These limited clusters do not strongly perturb the percolation of vacancies in GDC. The overall lower degree of defect clustering in GDC points to a more random and open defect landscape compared to NDC, which helps prevent strong localized trapping and supports more continuous oxygen ion migration. These structural characteristics are consistent with the well-established role of GDC as a high-performance ionic conductor. These findings align with those of Wei et al.^61^ and confirm the link between reduced clustering, increased randomness, and improved ionic transport in Gd-doped ceria. They are also supported by the early work of Butler et al., which identified Gd as exhibiting the weakest dopant–vacancy interactions among doped ceria systems.^18^

### 3.4 Vacancy Ordering

In the ideal cubic fluorite structure of doped ceria, oxygen vacancy pairs can align along the ⟨100⟩, ⟨110⟩, and ⟨111⟩ directions relative to the cation sublattice (Figure 4a).^62^ If vacancies are randomly distributed, their occurrence along these directions follows a geometric ratio of approximately 1:2:1.3, reflecting the relative number of equivalent neighbor pair directions available in the fluorite lattice.^62,63^ However, in doped ceria, deviations from this random distribution occur as vacancies associate with dopant ions,^59,64^ host cations,^65^ or other vacancies,^8,66^ forming various defect clusters. These clusters can hinder O^2–^ migration, suppressing ionic conductivity. The RMC analysis reveals clear deviations in vacancy pair ordering from a fully random distribution. As summarized in Table 2, GDC shows no preference for ⟨100⟩ vacancy pair alignment, favoring ⟨111⟩ and ⟨110⟩ instead, unlike NDC which prefers vacancy pair alignment along ⟨100⟩. Partial pair correlation functions *g*_*ij*_(*r*) further confirm these trends, with V_O_^••^–V_O_^••^ distributions deviating from those for O–O (effectively equivalent to a random distribution of vacancies on the O sites) in both systems (Figure 4b,c). The “noisier” V_O_^••^–V_O_^••^ signal in GDC (Figure 4b) may reflect the presence of nanodomains with local ordering resembling a mixture of fluorite and C-type Gd_2_O_3_ phases, as reported in previous total scattering studies,^71^ and recent in situ environmental TEM experiments, which observed oxygen vacancies in GDC can reversibly rearrange to form C-type motifs under electron beam irradiation.^67^ These nanodomains disrupt uniform vacancy alignment and lead to more variable local environments compared to the more consistently ordered NDC. Although our RMC–neutron analysis suggests heterogeneous local structures, including defect clustering and preferred vacancy directions, the configurations obtained are statistically averaged rather than directly imaging atomic-scale defects or their dynamics. Future studies employing advanced techniques such as in situ TEM and time-resolved diffraction would be excellent complementary approaches to directly visualize vacancy configurations, migration pathways and local heterogeneities.

**Figure 4:**
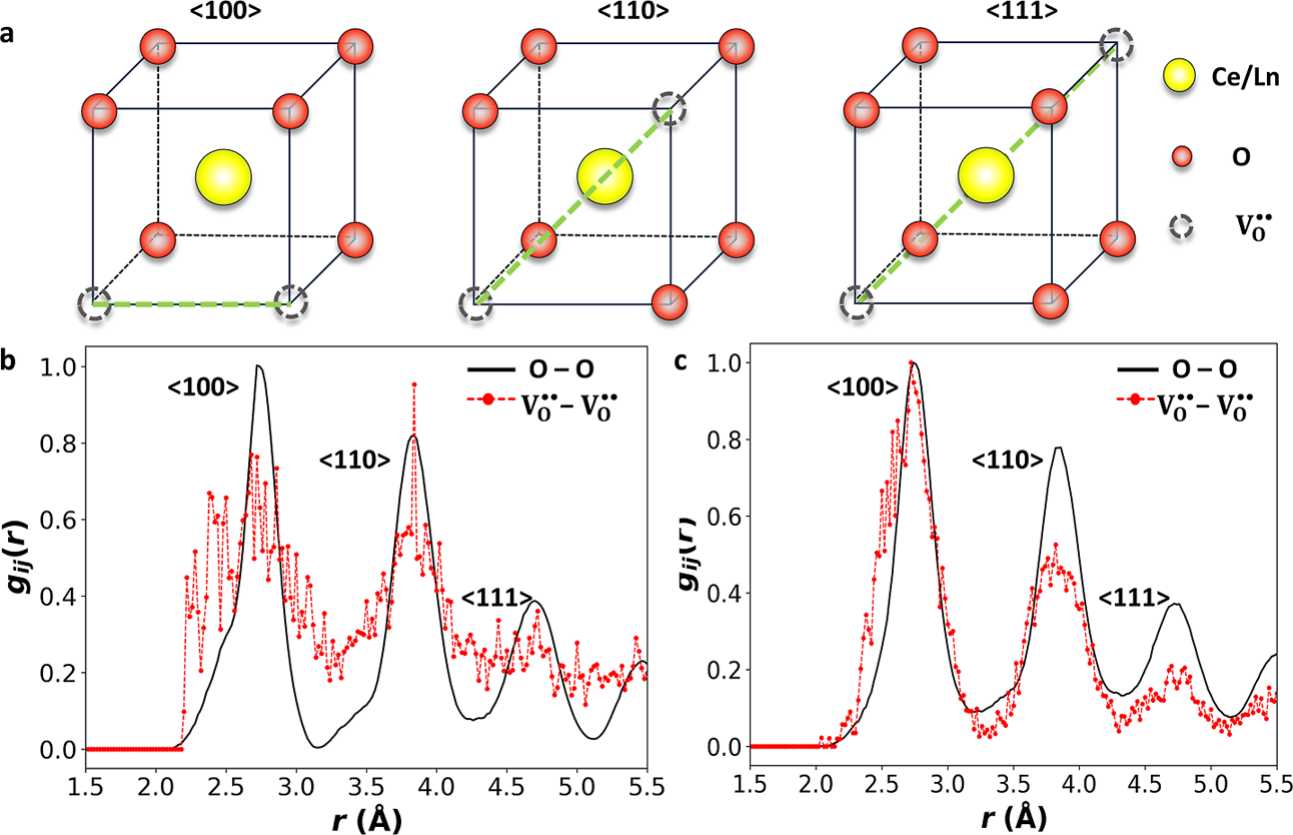
(a) Three possible alignments of oxide ion vacancy-pairs in the fluorite structure of doped ceria. *g*_OO_(*r*) and *g*_VV_(*r*) partial pair correlation functions for (b) GDC and (c) NDC averaged over 10 final RMC configurations in each case. The NDC data are adapted from ref 23 for comparison.

**Table 2:** Ratios of Vacancy Pairs in Different Alignments Derived from Final RMC configurations Compared to an Ideal Random Distribution of Vacancies in GDC and NDC^a^

vacancy pair	⟨100⟩	⟨110⟩	⟨111⟩	⟨100⟩/⟨110⟩	⟨100⟩/⟨111⟩
Ideal	1	2.0	1.3	0.5	0.77
GDC	1	2.32	1.57	0.78	0.65
NDC	1	1.12	0.65	0.89	1.56

a The NDC data are adapted from ref 23 for comparison.

The preference for ⟨100⟩ vacancy pair alignment in NDC contrasts with previous studies on Gd-, Sm-, Dy-, and Yb-doped ceria, using electron energy loss spectroscopy (EELS) and selected area electron diffraction (SAED), where 1/2 ⟨110⟩ ordering of vacancy pairs was reported, similar to that in C-type Ln_2_O_3_.^68^ A preference for ⟨111⟩ vacancy pair ordering in GDC has also been reported in DFT studies on GDC surfaces,^69^ while similar neutron diffraction studies on reduced ceria (CeO_2–*x*_),^70^ and Y-doped ceria,^71^ also find a preference for ⟨111⟩ vacancy pair alignment. The differences in local structure suggest that dopant type and defect–defect interactions have significant effects on the vacancy ordering patterns and with it the efficiency of ionic conduction in doped ceria systems. The observed experimental differences in defect properties between GDC and NDC point to the critical role of dopant size in shaping the vacancy ordering and conduction mechanisms in doped ceria.

### 3.5 Simulated Energy Distribution of Defect Configurations

While the structural and clustering trends in GDC and NDC provide a framework for understanding vacancy distribution and mobility, a deeper insight into the underlying mechanisms requires an examination of the energetics of defect formation. By correlating the calculated defect energy profiles with these experimental structural features, we further elucidate how dopant size and interactions influence conduction pathways in doped ceria systems. In undoped ceria, doubly charged oxygen vacancies (V_O_^••^) repel each other at close distances. The interaction energies of two vacancy pairs formed along the ⟨100⟩, ⟨110⟩, and ⟨111⟩ directions were calculated by the Mott–Littleton approach as 1.54, 0.69, and 0.61 eV, respectively (positive values indicate repulsive interaction). Notably, vacancy ordering in the ⟨100⟩ direction is the most strongly repulsive. Consequently, if no dopants are present, oxygen vacancies tend to remain distant rather than form closely spaced clusters, especially in the closest ⟨100⟩ alignment. However, our experimental observations clearly show nonrandom vacancy ordering in both GDC and NDC, indicating that the formation of vacancy clusters is stabilized by neighboring dopants.

To understand how dopant type affects the vacancy clustering, we calculated vacancy–dopant interactions using a newly developed high-throughput Mott–Littleton approach.^46^ All possible combinations of dopants occupying nearest-neighbor (NN) and next-nearest-neighbor (NNN) sites relative to two aligned vacancies in three directions were considered. First, charge-neutral defect complexes, [2 V_O_^••^–4Ln_Ce_^*’*^]^×^, were examined in both Nd- and Gd-doped ceria for vacancy pairs along the ⟨100⟩, ⟨110⟩, and ⟨111⟩ directions, including 7315, 10,626, and 12,650 possible configurations, respectively. Figure 5a,b show the density of states (DOS) of the calculated formation energies for all defect configurations. Importantly, the dopant distribution has a pronounced effect on the relative stability of the defect complexes, producing energy differences as large as 3–4 eV in Nd-doped ceria and 4–5 eV in Gd-doped ceria among various configurations. The lower calculated formation energies of the charge-neutral defect complexes in NDC indicate a greater overall tendency to form defect clusters, compared to those in GDC. This result is also in agreement with neutron scattering results, where NDC displays higher proportions of defect clustering than GDC, including Ln-V_O_^••^, V_O_^••^–V_O_^••^ and Ln–Ln (Figure 3c,f,i).

**Figure 5:**
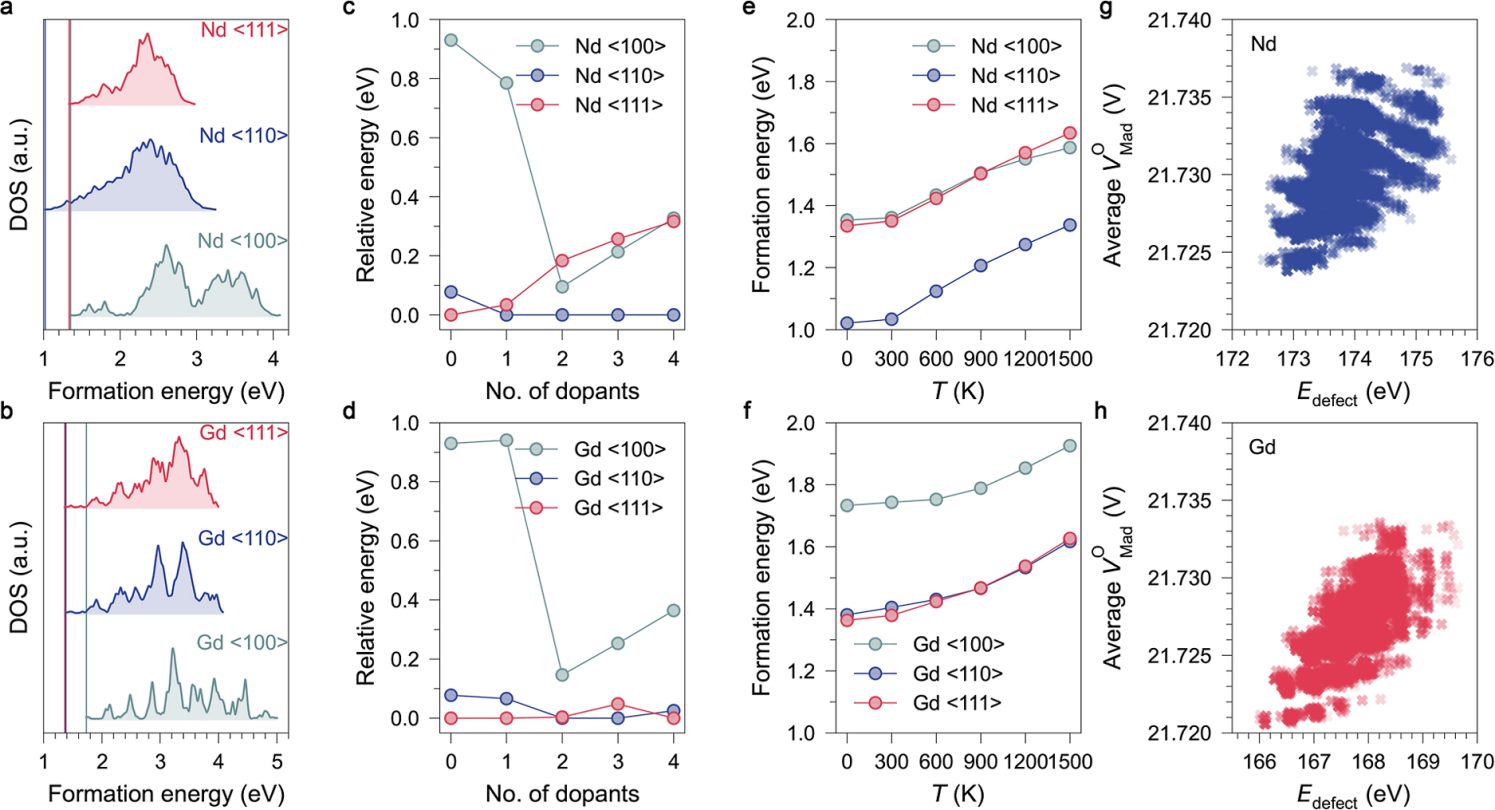
Comparison of the dopant-induced ordered oxygen vacancy pairs formed in Nd- and Gd-doped ceria. (a,b) The density of states (DOS) for the charge-neutral defect complexes, [2V_O_^••^–4Ln_Ce_^*’*^]^×^ in (a) Nd- and (b) Gd-doped ceria with vacancies aligned in the ⟨100⟩, ⟨110⟩, and ⟨111⟩ crystallographic directions. (c,d) The relative stability of oxygen vacancy pairs aligned along the three directions in (c) Nd- and (d) Gd-doped ceria, stabilized by varying numbers of dopant ions. (e,f) Temperature dependence of the formation energies of oxygen vacancy pairs along the three directions in the [2 V_O_^••^–4Ln_Ce_^*’*^]^×^ complexes in (e) Nd- and (f) Gd-doped ceria. (g,h) Average Madelung potentials on O sites (V) in the optimized Mott–Littleton defect models of [2V_O_^••^–4Ln_Ce_^*’*^]^×^ as a function of their corresponding defect energies (eV) for Nd-doped (blue) and Gd-doped (red) ceria.

In the Nd-doped system (Figure 5a), the minimum formation energy is 1.047 eV for vacancy pairs along ⟨110⟩, while the most stable ⟨100⟩ and ⟨111⟩ configurations have slightly higher energies (1.380 and 1.361 eV). In Gd-doped ceria (Figure 5b), the minimal energies for vacancy pairs along ⟨110⟩ and ⟨111⟩ are similar (1.381 and 1.363 eV), both lower than that along ⟨100⟩ (1.733 eV). The small energy differences among the most stable configurations explain the experimental observation of all three orientations of vacancy pairs. In both Nd- and Gd-doped ceria, the ⟨110⟩ alignment remains generally the most stable. Similar ⟨110⟩ vacancy ordering has also been observed in GDC through EELS and SAED studies, particularly at high dopant levels, where the local structure begins to resemble that of C-type Ln_2_O_3_ oxides.^68,72^

### 3.6 Stability Trends in Vacancy Configurations

At the macroscale in doped ceria, defects and defect clusters may not be charge-neutral and can electrostatically compensate one another. We therefore further consider other possible defect configurations. Table S7 shows the Mott–Littleton defect energies calculated for the most stable defect clusters containing between 0 and 4 dopants surrounding two vacancies along each of the three crystallographic directions. To compare trends in relative stability across orientations of vacancy pairs, we normalized defect energies to the lowest value in each scenario, as shown in Figure 5c,d. When one dopant ion is introduced, the configuration in which the dopant connects the two vacancies is the global minimum for all three directions (Figure 6a–f). The energy difference between the ⟨100⟩ and ⟨111⟩ alignments in Nd-doped ceria decreases from 0.93 to 0.75 eV, whereas including Gd has a negligible impact (0.94 eV). With the introduction of more dopants, this trend persists: in Nd-doped ceria, two dopants make the ⟨100⟩-aligned vacancy pairs more stable than the ⟨111⟩ configuration, and only 0.1 eV less stable than the most stable ⟨110⟩ arrangement. Adding three and four Nd dopants increases this maximum difference by only 0.2 and 0.3 eV. Such small energy differences explain the occurrence of ⟨100⟩-aligned vacancy pairs observed experimentally in NDC.^23^ In contrast, in Gd-doped ceria (Figure 5d), the ⟨100⟩-aligned vacancy pairs never surpass the stability of ⟨111⟩ and ⟨110⟩ pair alignments, regardless of dopant concentration, in good agreement with our neutron scattering data. Nevertheless, the reduced energy difference between vacancy pairs along ⟨100⟩ and other directions due to Gd doping helps explain the partial ⟨100⟩ vacancy alignment observed in neutron scattering experiments on GDC.

**Figure 6:**
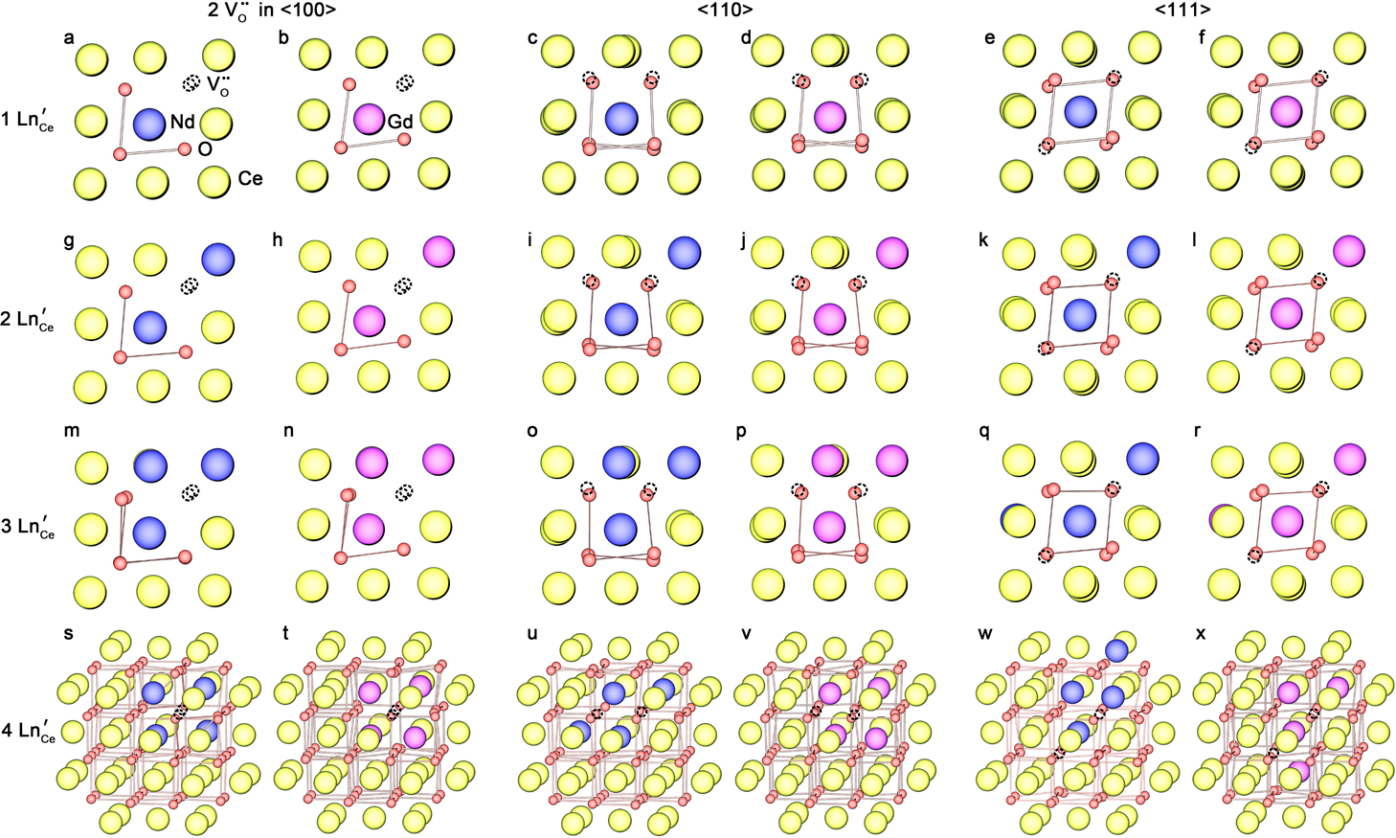
Lowest-energy configurations of defect clusters containing two oxygen vacancies and (a–f) 1, (g–l) 2, (m–r) 3, and (s–x) 4 Ln dopants in Nd- and Gd-doped ceria, identified by high-throughput Mott–Littleton calculations. The configurations are grouped by oxygen vacancy alignment: the first two columns show configurations with vacancies aligned along the ⟨100⟩ direction, the third and fourth columns correspond to ⟨110⟩, and the last two columns correspond to ⟨111⟩. Ce, O, Nd, Gd, and vacancy sites are shown in yellow, red, blue, magenta, and dashed circles, respectively.

The difference between Gd- and Nd-doped ceria is attributed to their ionic radii. With Ce^4+^ at 0.97 Å in 8-fold coordination with oxygen,^18^ Gd^3+^ (1.053 Å) is accommodated with less strain into the CeO_2_ lattice, causing less distortion than the larger Nd^3+^ (1.109 Å).^19,56,73^ This weaker distortion in Gd-doped ceria makes electrostatic interactions dominant, favoring ⟨110⟩ and ⟨111⟩ alignments where the vacancy separation is larger. In contrast, Nd^3+^, with a larger radius, induces greater distortion in CeO_2_, increasing Nd–O bond lengths (2.465 Å) compared to Gd–O (2.418 Å) and Ce–O (2.336 Å). This lattice expansion provides more room for ionic relaxation, easing electrostatic repulsion in the shortest-distance ⟨100⟩ vacancy configurations.^74^ As a result, ⟨100⟩-aligned vacancies can achieve comparable stability in Nd-doped ceria, unlike undoped ceria or with Gd doping. Overall, the stability of oxygen vacancy alignment depends on the relative ionic size of the dopant, affecting the balance of electrostatics and strain effects that determine the mechanism of defect clustering.

### 3.7 Lowest-Energy Defect Configurations

The lowest-energy configurations after structural optimization for vacancy pairs aligned along ⟨100⟩, ⟨110⟩, and ⟨111⟩ are shown in Figure 6. For one to three dopants, both Nd- and Gd-doped ceria show local structures that deviate from CeO_2_ toward a Ln_2_O_3_-like coordination (Figure 6a–r), with a slight difference in bond length, consistent with previous findings.^68,73,75^ The dopants predominantly occupy nearest-neighbor sites relative to at least one V_O_^••^ or both V_O_^••^, in order to maximize the V_O_^••^–Ln_Ce_^*’*^ association (denoted as NN_V_O_^••^); meanwhile, the dopant ions themselves tend to remain nearest neighbors to each other (denoted as NN_Ln). Together, these tendencies result in the formation of compact dopant–vacancy clusters. With four dopants, however, Nd- and Gd-doped ceria stabilize distinct configurations (Figure 6s–x). Along ⟨100⟩ in NDC, both vacancies are coordinated by three Nd^3+^ ions, forming a compact structure comparable to Nd_2_O_3_-like arrangements (Figure 6s). This configuration reduces the energy difference between ⟨100⟩ and other directions, allowing ⟨100⟩-aligned vacancy pairs to form with relative ease. In GDC, a different arrangement is observed: all Gd^3+^ ions tend to cluster around only one vacancy site (Figure 6t). A similar scenario holds for ⟨110⟩ alignments, where Nd^3+^ ions remain closely associated with both vacancies (Figure 6u), while Gd^3+^ dopants cluster near one vacancy (Figure 6v). This result aligns with previous computational results by Li et al.^76^ For ⟨111⟩-aligned vacancies, two Nd^3+^ ions remain at the next-nearest sites relative to one vacancy (NNN_V_O_^••^), remaining distant from the second vacancy (Figure 6w), while Gd^3+^ ions contact both vacancies, but adopt a more spatially extended configuration, resulting in a less compact arrangement (Figure 6x).

The arrangement of Nd^3+^ dopants near the vacancies aligned along both the ⟨100⟩ and ⟨110⟩ directions maximizes the V_O_^••^–Nd_Ce_^*’*^ interaction, forming compact configurations similar to those proposed by Li et al.^77^ Such a strong interaction is expected to reduce the mobility of vacancies, thereby decreasing the number of mobile vacancies contributing to ionic conductivity.^9^ As oxygen vacancies in ceria diffuse along the ⟨100⟩ direction, the presence of large Nd^3+^ dopants may permit a more flexible vacancy dynamic pattern with a less constrained ⟨111⟩ arrangement or isolated vacancies. This observation agrees with the energy trend shown in Figure 5c, where the introduction of two dopants leads to more stable defect clusters along ⟨100⟩ rather than ⟨111⟩. In GDC, Gd–Gd interactions dominate, encouraging denser Gd clusters. As a result, at least one of the two vacancies becomes less strongly attracted by Gd dopants (Figure 6t,v,x), yielding a more open environment for oxygen transport along the ⟨100⟩ direction. This dispersed configuration forms quasicontinuous migration pathways in GDC that enhance ionic conductivity, compared to systems featuring more compact vacancy clusters, with spatial confinement caused by larger dopant ions as seen in NDC.^8^

The difference in the V_O_^••^–Ln_Ce_^*’*^ interactions between Nd- and Gd-doped systems correlate with the maximum conductivity and doping levels observed experimentally. In Nd-doped ceria, the maximum low-temperature conductivity (σ_300_ (max) = 4.7 × 10^–6^ S cm^–1^) is achieved at *x* = 0.2, while the maximum high-temperature conductivity (σ_800_ (max) = 3.8 × 10^–2^ S cm^–1^) occurs at *x* = 0.15.^23^ In Gd-doped ceria, the maximum conductivity at both low and high temperatures is achieved at *x* = 0.2, yielding values of σ_300_ (max) = 6.6 × 10^–5^ S cm^–1^ and σ_800_ (max) = 8.7 × 10^–2^ S cm^–1^. With increasing dopant concentration, the formation of closely bound vacancy clusters is expected to become more pronounced due to the increased defect density. Previous Monte Carlo simulations of Y^3+^ doped CeO_2_ have shown that although higher Y_2_O_3_ doping levels enhance the overall vacancy concentration, they also lead to deep vacancy traps around Y^3+^ and significantly higher vacancy migration barriers (∼1.5–2.0 eV), ultimately hindering vacancy transport compared to dilute doping with a barrier of ∼0.4–0.6 eV.^10^ In NDC, compact defect clusters, such as the ⟨100⟩-aligned vacancies, form more easily than those in GDC, and may explain why the maximum conductivity is achieved in NDC at a lower dopant concentration.

Overall, the differences in defect configurations and ionic conductivity between GDC and NDC can be understood in terms of how the ionic size of the dopant tailors the defect chemistry of doped ceria systems. Nd^3+^, with a larger ionic size, reduces the local strain for forming ⟨100⟩-aligned vacancy pairs but creates energy barriers that hinder vacancy migration. In contrast, Gd^3+^, with a small size, creates a defect landscape with less pronounced vacancy aggregation that minimizes barriers for vacancy migration and provides a superior conduction pathway. This distinction highlights a critical size threshold in the lanthanide series, with Gd^3+^ acting as an ionic size boundary, as first shown by Butler et al.^18^ Dopants much larger than Gd^3+^, such as La^3+^, induce significant lattice distortions and suppress conductivity, while those close to Gd^3+^, such as Sm^3+^ or Eu^3+^, balance lattice expansion with reduced clustering, enhancing ionic conductivity.^9,17,78^ Conversely, dopants smaller than Gd^3+^, like Sc^3+^, Tm^3+^ and Yb^3+^, can shrink the lattice or induce phase transitions, and their tendency to relax toward nearby vacancies stabilizes defect complexes, thereby restricting conduction pathways for vacancy movement.^18,79,80^

### 3.8 Temperature and Electrostatic Effects on Defect Structure

Although the neutron experiments on GDC were performed at room temperature, the simulations incorporate temperature effects, enabling us to assess the evolution of defect configurations under SOFC-relevant conditions. At elevated temperatures, defect complexes tend to dissociate,^15,37^ and therefore we considered temperature effects by including configurational entropy and evaluating the statistical occupation probabilities of defect configurations based on eqs 4 and 5. With increasing temperature, less stable configurations with energies close to the ground state play more significant roles in the ensemble. We observed a slight shift of the relative stability between the ⟨100⟩ and ⟨111⟩-aligned [2V_O_^••^–4Ln_Ce_^*’*^]^×^ configurations in NDC (Figure 5e), as well as between ⟨110⟩ and ⟨111⟩ aligned configurations in Gd-doped ceria (Figure 5f). For NDC, as the temperature increases, the formation energy of vacancy pairs along the ⟨100⟩ direction decreases below that of ⟨111⟩ aligned pairs near 900 K, making the ⟨100⟩ direction eventually more stable at higher temperatures. In this study, the observed trend agrees with earlier neutron results on Nd-doped ceria, where ⟨100⟩ vacancy clustering decreased slightly at elevated temperature (600 °C) relative to room temperature.^23^ In GDC, the ⟨100⟩ direction consistently exhibits the highest energy throughout the studied temperature range (0–1500 K), indicating inherently lower stability of ⟨100⟩-aligned clustering, consistent with our experimental results showing that the ⟨100⟩ vacancy pairs in Gd-doped ceria tend to be least favorable. Above 800 K, the ⟨110⟩ aligned vacancy clusters become more stable than those along the ⟨111⟩ direction. High-temperature neutron diffraction studies on reduced CeO_2_ at high defect concentrations (up to 1273 K)^70^ and on δ-Bi_2_O_3_ (up to ∼1033 K)^81^ have also shown that short-range oxygen–vacancy correlations and distorted local environments persist at SOFC-relevant temperatures in fluorite-type oxides. These findings support that our theoretical predictions, which show minimal variation in defect configurations between room and SOFC-operational temperature ranges.

In ionic solids, Madelung potentials will be affected by both the effective valence and the crystal structure.^82^ The Madelung potential quantifies the Coulombic potential at ionic sites with contributions from all other charged ions in the lattice, serving as an effective descriptor for many macroscopic properties in metal oxides.^83^ For example, a linear relationship has been identified between Madelung potentials and absolute band edge positions in metal oxides.^48,84^ Figure 5g,h shows the average Madelung potentials on O sites (V_Mad_^O^) in all optimized configurations of the [2V_O_^••^–4Ln_Ce_^*’*^]^×^ defect complexes as a function of their corresponding defect energies. In general, the Gd-doped systems have slightly lower V_Mad_^O^ compared to Nd-doped systems, indicating weaker electrostatic attraction from cations. Furthermore, configurations with lower average Madelung potentials on O sites generally correspond to lower defect energies in both systems, suggesting that more stable defect complexes correspond to weaker electrostatic fields. Such weakened electrostatic interactions in turn lower the migration barrier for oxygen ions and elevate ionic conductivity, particularly in Gd-doped ceria.

## 4 Conclusions

By bringing together experimental neutron data and high-throughput atomistic simulations, this study provides a comprehensive understanding of how lanthanide dopant size controls the defect chemistry of doped ceria systems. There is excellent consistency between experimental and simulation results which affirms the distinct directional vacancy preferences, reinforcing the role of dopant size in shaping defect structure and transport behavior. Gd^3+^ is an optimal dopant, with its intermediate size achieving the right balance between lattice expansion, defect clustering, and oxygen vacancy mobility. Dopants outside of the Nd^3+^ to Gd^3+^ range, whether larger or smaller, tend to deviate from this optimal behavior, reducing their effectiveness in promoting efficient ionic transport. This study demonstrates that rational selection of dopant ionic size can control vacancy ordering and defect energetics, providing guidance for designing ceria-based electrolytes with optimized ionic conduction properties for SOFC applications. The methodologies and principles outlined in this study could also be extended to other doped systems and functional materials aimed at advancing clean technologies.

## Supplementary Material



## Notes

Neutron and X-ray diffraction refinement data for Gd-doped ceria; coordination number and pair distribution functions from RMC fitting results; newly fitted interatomic potentials for Nd_2_O_3_; computational parameters used in Mott–Littleton simulations; and calculated defect formation energies for Nd- and Gd-doped ceria (PDF)
