# Supplementary material for: Probing Dopant Size Effects on Defect Clustering and Vacancy Ordering in Lanthanide-doped Ceria
Source: J Am Chem Soc. 2025 Aug 21;147(35):31992–2004. doi: 10.1021/jacs.5c09862 (PMC12412159; doi:10.1021/jacs.5c09862)
Supplement: Supplementary file 1 [file ja5c09862_si_001.pdf]

Probing Dopant Size Effects on Defect Clustering and Vacancy Ordering in Lanthanide-doped Ceria

Jing Ming,[1‡] Xingfan Zhang,[2‡] Marzena Leszczyńska-Redek,[3*] Marcin Malys,[3] Maciej Wojcik,[3] Wojciech Wrobel,[3] Stephen Hull,[4] Franciszek Krok,[3] Woongkyu Jee,[2] Marcin Krynski,[3] Alexey A. Sokol,[2] Scott M. Woodley,[2] C. Richard A. Catlow,[2,5*] and Isaac Abrahams[1*]

[1]Department of Chemistry, Queen Mary University of London, Mile End Road, London E1 4NS, U.K.

[2]Kathleen Lonsdale Materials Chemistry, Department of Chemistry, University College London, London WC1H 0AJ, U.K.

[3]Faculty of Physics, Warsaw University of Technology, Koszykowa 75, 00-662 Warszawa, Poland

[4]STFC ISIS Facility, Rutherford Appleton Laboratory, Chilton, Didcot, Oxon OX11 0QX, U.K.

[5]School of Chemistry, Cardiff University, Park Place, Cardiff CF10 1AT, U.K.

[‡]*These authors contributed equally to this work.*

[*]*Corresponding authors:*

Marzena Leszczyńska-Redek Email: marzena.redek@pw.edu.pl
Isaac Abrahams Email: i.abrahams@qmul.ac.uk
Richard A. Catlow Email: c.r.a.catlow@ucl.ac.uk

Table S1. Crystal and refinement parameters for GDC.

**Formula (nominal)**	$Ce_{0.8}{}^{160}Gd_{0.2}O_{1.9}$	**No of profile points**	Neut bs	4522
**Temp (ºC)**	20		Neut 90°	2522
**Formula weight (g*mol^{-1})**	174.194		X-ray	6762
**Space Group**	*Fm-3m*	**No. of reflections**	Neut bs	271
Z	4		Neut 90°	242
**D_{calc} (g*cm^{-3})**	7.244		X-ray	30
***a* (Å)**	5.42576(3)	**No. of variables**	104	
**Volume (Å^3)**	159.728(3)			

**R-Factors**					
**Neut bs**	R_{wp}	0.0295	**X-ray**	R_{wp}	0.0941
	R_p	0.0386		R_p	0.0728
	R_{ex}	0.0142		R_{ex}	0.0537
	R_{F^2}	0.2161		R_{F^2}	0.1098
**Neut. 90°**	R_{wp}	0.0311	**Totals**	R_{wp}	0.0356
	R_p	0.0342		R_p	0.0720
	R_{ex}	0.0091			
	R_{F^2}	0.2332			

Table S2. Refined structural parameters for GDC at room temperature.

**Atom**	**Site**	***x***	***y***	***z***	**Occ.**	***U*$_{iso}$ (Å^2)**
$Ce_{0.8}{}^{160}Gd_{0.2}O_{1.9}$						
Ce	*4a*	0.0	0.0	0.0	0.800	0.00422(6)
^{160}Gd	*4a*	0.0	0.0	0.0	0.196	0.00422(6)
Gd	*4a*	0.0	0.0	0.0	0.004	0.00422(6)
O	*8c*	0.25	0.25	0.25	0.941(2)	0.00814(6)

Table S3. Nearest-neighbor “coordination numbers” for different atom/vacancy ($V_O^{\bullet\bullet}$) pairs in the system GDC and NDC. Data for NDC are taken from Ming et al.[a]

Atom Pairs	**GDC**	**NDC**	**Random**
Ce-Ce	9.75(3)	9.89(7)	9.6
Ce-Gd/Nd	2.26(3)	2.11(7)	2.4
Ce-O	7.742(8)	7.755(7)	7.6
Ce-$V_O^{\bullet\bullet}$	0.300(8)	0.251(8)	0.4
Gd/Nd-Ce	9.03(12)	8.44(29)	9.6
Gd-Gd/Nd-Nd	2.99(12)	3.57(28)	2.4
Gd/Nd-O	7.33(3)	7.03(4)	7.6
Gd/Nd-$V_O^{\bullet\bullet}$	0.66(3)	0.99(3)	0.4
O-Ce	3.260(3)	3.265(3)	3.2
O-Gd/Nd	0.768(2)	0.740(4)	0.8
O-O	5.783(4)	6.098(7)	5.7
O-$V_O^{\bullet\bullet}$	0.273(4)	0.268(4)	0.3
$V_O^{\bullet\bullet}$-O	5.20(8)	5.11(7)	5.7
$V_O^{\bullet\bullet}$-$V_O^{\bullet\bullet}$	0.56(8)	0.91(7)	0.3

[a] Ming, J.; Leszczyńska-Redek, M.; Malys, M.; Wrobel, W.; Jamroz, J.; Struzik, M.; Hull, S.; Krok, F.; Abrahams, I. Dopant Clustering and Vacancy Ordering in Neodymium Doped Ceria. *J. Mater. Chem. A* **2024**, *12* (17), 10203–10215. https://doi.org/10.1039/D3TA07668G.

Table S4. Percentage of nearest neighbor distributions for different atom/vacancy ($V_O^{\bullet\bullet}$) pairs from final RMC configurations in the GDC and NDC system (*x* = 0.2) compared to the values for fully random distributions (*Ran.*). Data for NDC are taken from Ming et al.[a] Bold highlights dopant-dopant, dopant-$V_O^{\bullet\bullet}$ and $V_O^{\bullet\bullet}$-$V_O^{\bullet\bullet}$ in each system for comparison.

GDC	Ln-Ln	Ce-Ce	Ce-Gd	Gd-Ce	Gd-Gd	*Ran.*
		81.2(3)	18.8(3)	75(1)	**24(1)**	80 : 20
	Ln-O/$V_O^{\bullet\bullet}$	Ce-O	Ce-$V_O^{\bullet\bullet}$	Gd-O	Gd-$V_O^{\bullet\bullet}$	*Ran.*
		96.33(7)	3.67(7)	91.7(2)	**8.3(2)**	95 : 5
	O/$V_O^{\bullet\bullet}$ -$V_O^{\bullet\bullet}$/O	O-O	O-$V_O^{\bullet\bullet}$	$V_O^{\bullet\bullet}$-O	$V_O^{\bullet\bullet}$-$V_O^{\bullet\bullet}$	*Ran.*
		95.50(7)	4.50(7)	90.3(8)	**9.7(7)**	95 : 5
NDC	Ln-Ln	Ce-Ce	Ce-Nd	Gd-Ce	Nd-Nd	*Ran.*
		82.4(6)	17.6(6)	70(2)	**30(2)**	80 : 20
	Ln-O/$V_O^{\bullet\bullet}$	Ce-O	Ce-$V_O^{\bullet\bullet}$	Nd-O	Nd-$V_O^{\bullet\bullet}$	*Ran.*
		96.9(1)	3.1(1)	87.6(4)	**12.4(4)**	95 : 5
	O/$V_O^{\bullet\bullet}$ -$V_O^{\bullet\bullet}$/O	O-O	O-$V_O^{\bullet\bullet}$	$V_O^{\bullet\bullet}$-O	$V_O^{\bullet\bullet}$-$V_O^{\bullet\bullet}$	*Ran.*
		95.78(5)	4.22(5)	85(1)	**15(1)**	95 : 5

[a] Ming, J.; Leszczyńska-Redek, M.; Malys, M.; Wrobel, W.; Jamroz, J.; Struzik, M.; Hull, S.; Krok, F.; Abrahams, I. Dopant Clustering and Vacancy Ordering in Neodymium Doped Ceria. *J. Mater. Chem. A* **2024**, *12* (17), 10203–10215. https://doi.org/10.1039/D3TA07668G.

Table S5. The performance of the newly fitted potential for reproducing the structures and properties of *C*-Nd_2O_3 and *A*-Nd_2O_3 compared to experimental and DFT references. Observables include lattice constants (a_0, c_0), static and high-frequency dielectric constants (ε_0 and ε_∞), elastic constants (C_{11}, C_{12}, and C_{44}), and bulk modulus (B_0).

Observables	Shell Model Prediction	Ref. (Expt.)	Ref. (DFT)
(a) *C*-type Nd_2O_3			
a_0 (Å)	11.109	11.102[a]	11.176[b]
ε_0	16.35	13.6-15.4[c]	
ε_∞	4.177	3.725[c]	
C_{11} (GPa)	214.03		179[d]
C_{12} (GPa)	102.81		97[d]
C_{44} (GPa)	55.44		58[d]
B_0 (GPa)	139.89		124[d]
(b) *A*-type Nd_2O_3			
a_0 (Å)	3.851	3.841[a]	3.859[b]
c_0 (Å)	6.084	6.027[a]	6.072[b]
ε_0	19.03	17-21[c]	
ε_∞	4.41	4.452[c]	
B_0 (GPa)	155.31	142[e]	

[a] Artini, C.; Pani, M.; Plaisier, J. R.; Costa, G. A. Structural Study of Nd Oxidation by Means of In-Situ Synchrotron X-Ray Diffraction (400°C ≤ T ≤ 700°C). *Solid State Ionics* **2014**, *257*, 38–41. https://doi.org/10.1016/j.ssi.2014.01.034.
[b] Hirosaki, N.; Ogata, S.; Kocer, C. Ab Initio Calculation of the Crystal Structure of the Lanthanide Ln_2O_3 Sesquioxides. *Journal of Alloys and Compounds* **2003**, *351* (1), 31–34. https://doi.org/10.1016/S0925-8388(02)01043-5.
[c] Busani, T.; Devine, R.; Gonon, P. Structural Effects in the Dielectric Constant of Rare-Earth Oxides: Nd_2O_3. *ECS Trans.* **2006**, *1* (5), 331. https://doi.org/10.1149/1.2209282.
[d] De Jong, M.; Chen, W.; Angsten, T.; Jain, A.; Notestine, R.; Gamst, A.; Sluiter, M.; Krishna Ande, C.; van der Zwaag, S.; Plata, J. J.; Toher, C.; Curtarolo, S.; Ceder, G.; Persson, K. A.; Asta, M. Charting the Complete Elastic Properties of Inorganic Crystalline Compounds. *Sci Data* **2015**, *2* (1), 150009. https://doi.org/10.1038/sdata.2015.9.
[e] Jiang, S.; Liu, J.; Bai, L.; Li, X.; Li, Y.; He, S.; Yan, S.; Liang, D. Anomalous Compression Behavior in Nd_2O_3 Studied by X-Ray Diffraction and Raman Spectroscopy. *AIP Advances* **2018**, *8* (2), 025019. https://doi.org/10.1063/1.5018020.

Table S6. GULP-readable format of the set of shell-model potentials used in this work.

```
species
Ce  core  -9.850000
Ce  shel  13.850000
La  core  -9.21790
La  shell  12.21790
O   core  0.936345
O   shell  -2.936345
Nd  core -10.533239
Nd  shell  13.533239
Gd  core  3.0
buck
O  shell O  shell 22764.3000  0.149000 20.983768   0.0 25.0
O  shell Ce  shell 1139.010286 0.417578 25.082349   0.0 4.8
O  shell La  shell 2248.3906  0.341000 34.477930   0.0 25.0
Ce  shell Ce  shell 1.0     0.10   30.481293   0.0 25.0
La  shell La  shell 1.0     0.10   56.693757   0.0 25.0
La  shell Ce  shell 1.0     0.10   39.621401   0.0 25.0
Gd  core O  shell 1336.8    0.3551  0.0      0.0 25.0
Nd  shell O  shell 1708.4227  0.355000 30.000000   0.0 25.0
Nd  shell Nd  shell 1.0     0.10   50.000000   0.0 25.0
Nd  shell Ce  shell 1.0     0.10   39.621401   0.0 25.0
lennard 12 6
O  shell O  shell 10.0   0.0   0.0 25.0
O  shell Ce  shell 10.0   0.0   0.0 4.8
O  shell La  shell 10.0   0.0   0.0 25.0
Ce  shell Ce  shell 10.0   0.0   0.0 25.0
La  shell La  shell 10.0   0.0   0.0 25.0
La  shell Ce  shell 10.0   0.0   0.0 25.0
Nd  shell Nd  shell 10.0   0.0   0.0 25.0
Nd  shell Ce  shell 10.0   0.0   0.0 25.0
O  shell Nd  shell 10.0   0.0   0.0 25.0
morse
O  shell Ce  shell -1.15172262 0.40 4.53327 0.0000 0.000  4.80
polynomial
 1
O  shell Ce  shell -1.077769  0.00 0.000 0.000  4.80
polynomial
 5
O  shell Ce  shell -1952.35663530 1796.71132884 -658.98890407 &
120.42275309 -10.96459980 0.39794942 0.000 4.8 5.5
spring
Ce  1071.1845   100000.0
La  799.14006   119228.97
Nd  1154.9222   100000.00
O   53.022513   50000.0
```

Table S7. Defect energies (eV) calculated by the Mott-Littleton approach for different dopant-vacancy clusters, with vacancy pairs aligned in different directions in Nd- and Gd-doped ceria. The most stable pattern in each system is in bold.

	No. of dopants	<100>	<110>	<111>
Nd-doped	0	33.62	32.77	**32.69**
	1	67.84	**67.05**	67.09
	2	102.18	**102.09**	102.27
	3	137.49	**137.28**	137.54
	4	172.85	**172.52**	172.84
Gd-doped	0	33.62	32.77	**32.69**
	1	66.25	65.38	**65.31**
	2	98.98	**98.83**	98.84
	3	132.66	**132.41**	132.45
	4	166.38	166.05	**166.02**

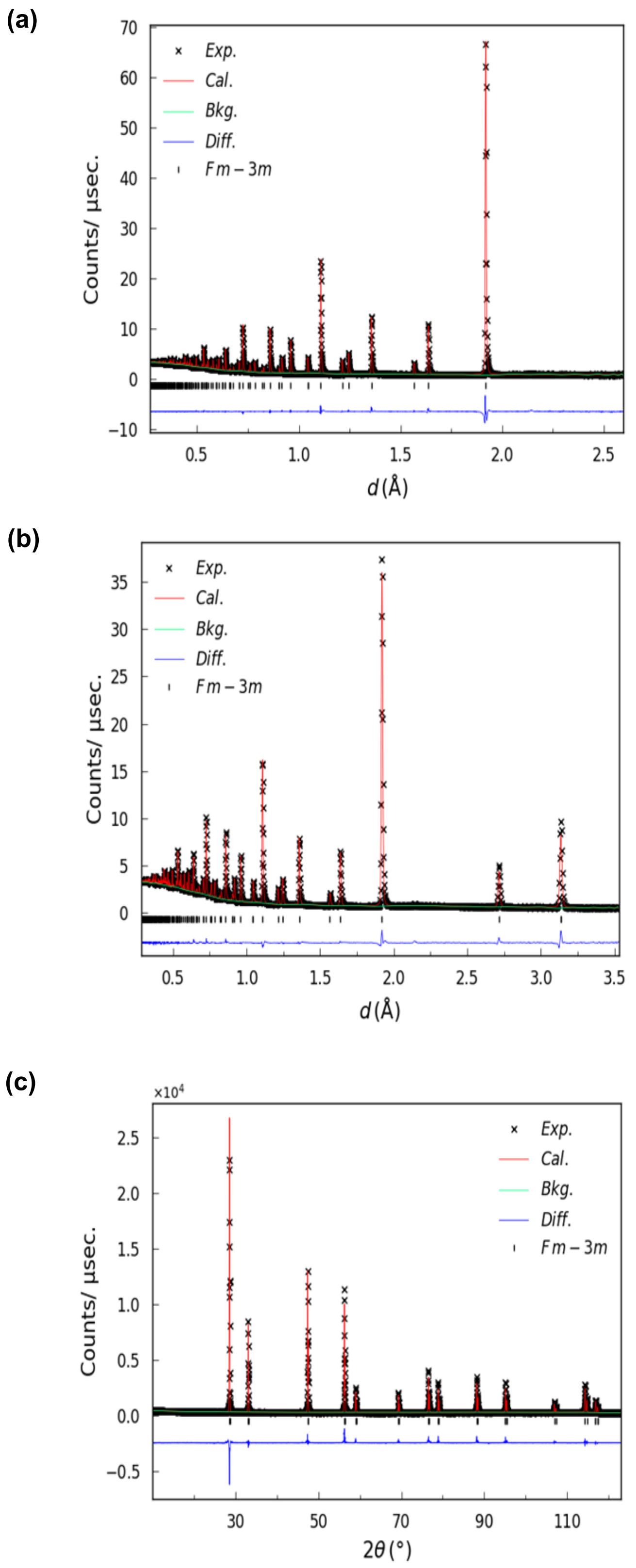

Figure S1. Fitted diffraction profiles for GDC at room temperature using the $Fm\overline{3}m$ model showing fits to (a) neutron backscattering, (b) neutron 90º data and (c) XRD data.

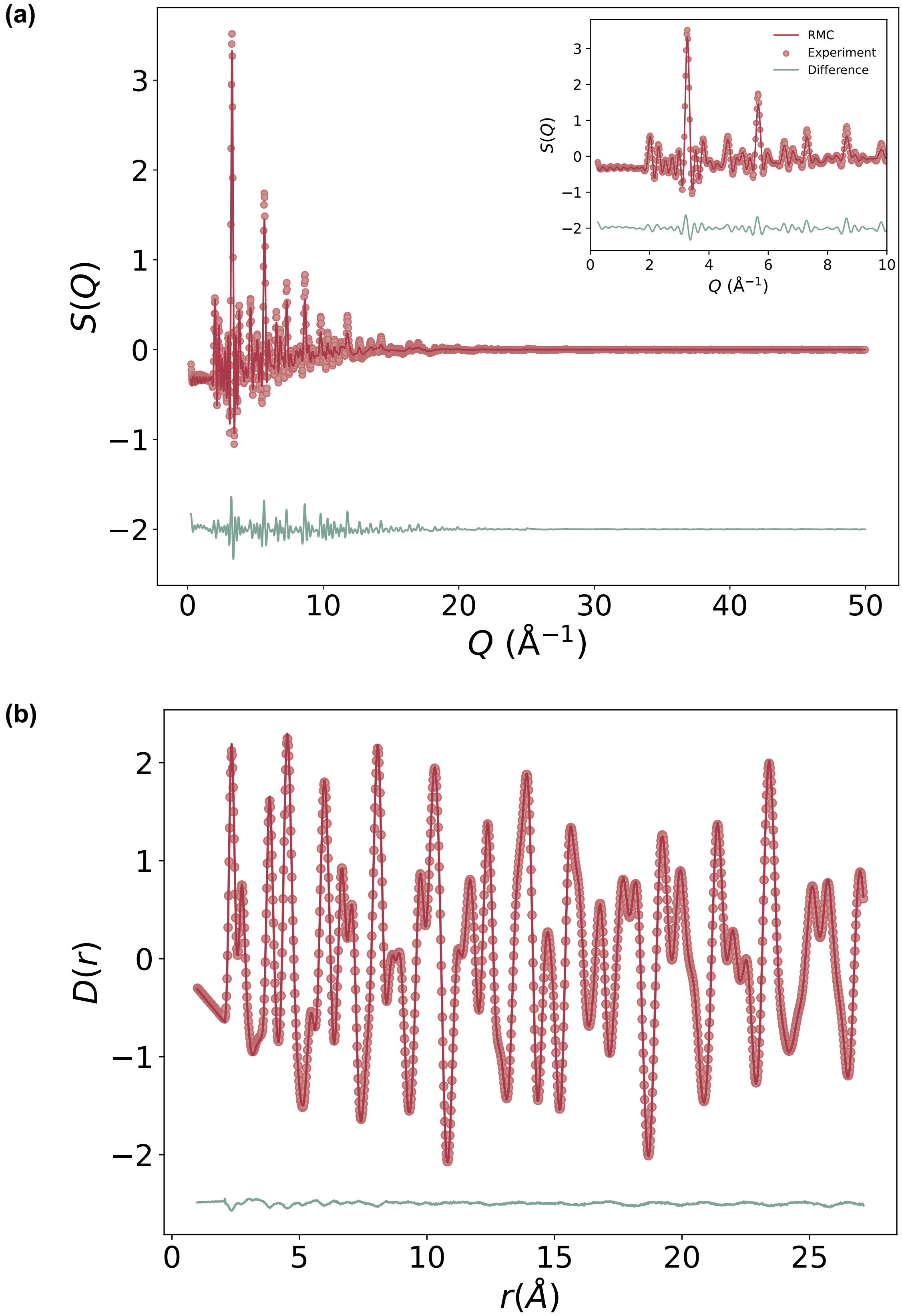

Figure S2. Fitted normalized (a) total scattering structure factor S(*Q*) with an inset highlighting the enlarged fit details at low, and (b) differential correlation function *D*(*r*) for GDC. The experimental data (red dots), calculated profile (black line), and difference (blue line) are presented.

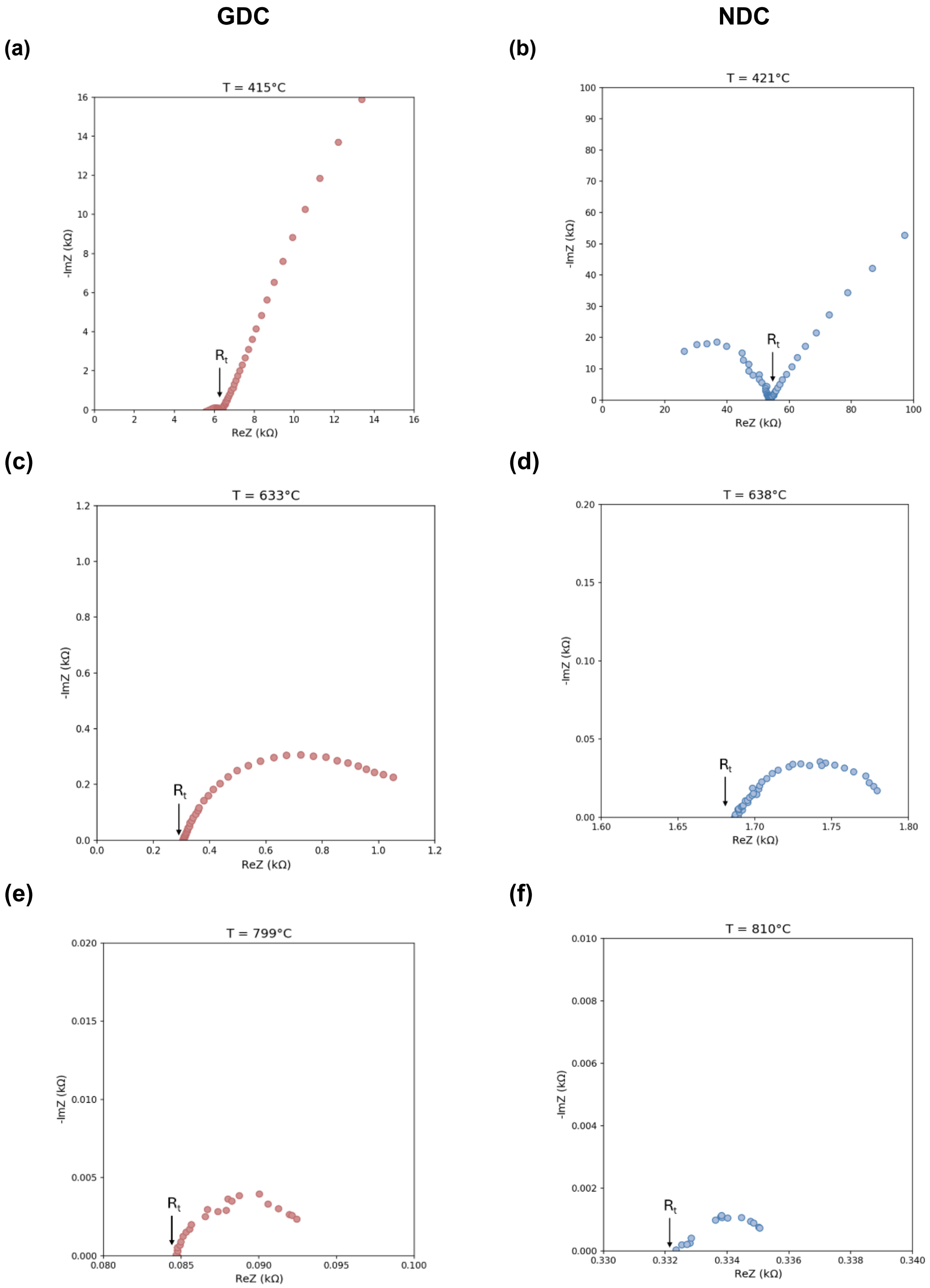

Figure S3. Nyquist plots of impedance spectra for GDC (red, a, c, e) and NDC (blue, b, d, f) at selected temperatures. The real part of impedance (ReZ) is plotted on the *x*-axis and the negative imaginary part (−ImZ) on the *y*-axis. The total resistance (R_t) is indicated on each plot by an arrow.
